# Supplementary material for: mAb14, a Monoclonal Antibody against Cell Surface PCNA: A Potential Tool for Sezary Syndrome Diagnosis and Targeted Immunotherapy
Source: Cancers (Basel). 2023 Sep 4;15(17):4421. doi: 10.3390/cancers15174421 (PMC10486495; doi:10.3390/cancers15174421)
Supplement: Supplementary file 1 [file cancers-15-04421-s001.zip › cancers-2480312-supplementary.docx]

| **SS patient** | **Gender** | **Age (years)** | **%CD4+CD26-** | **Total CD4 (cells/ul)** |
| --- | --- | --- | --- | --- |
| SS-9 | Female | 60 | 58% | 1392 |
| SS-14 | Female | 60 | 75% | 4563 |
| SS-15 | Female | 70 | 56% | 3000 |
| SS-16 | Male | 38 | 58% | 1554 |
| SS-17 | Male | 76 | 36% | 1515 |
| SS-18 | Female | 60 | 45% | 1608 |
| SS-19 | Male | 52 | 55% | 391 |
| SS-20 | Female | 88 | 48% | 1381 |
| SS-21 | Female | 82 | 79% | 3500 |

**Table S1. Clinical data of Sezary patients.**


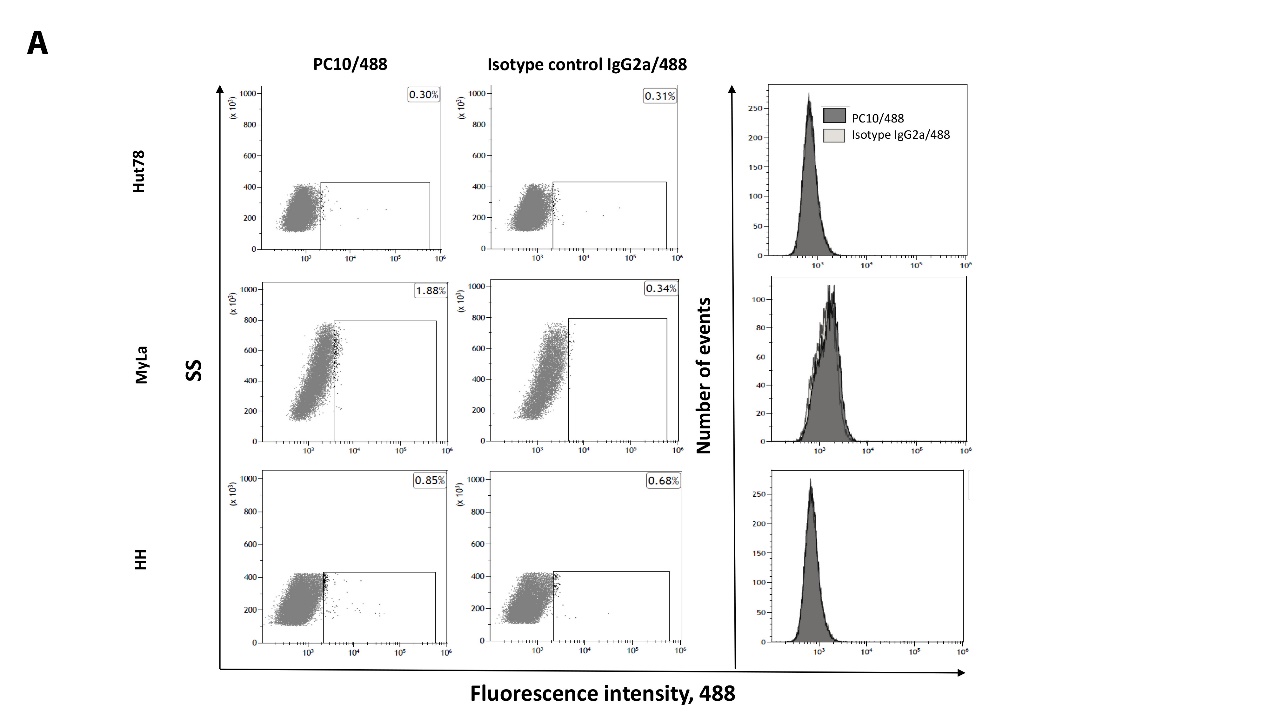


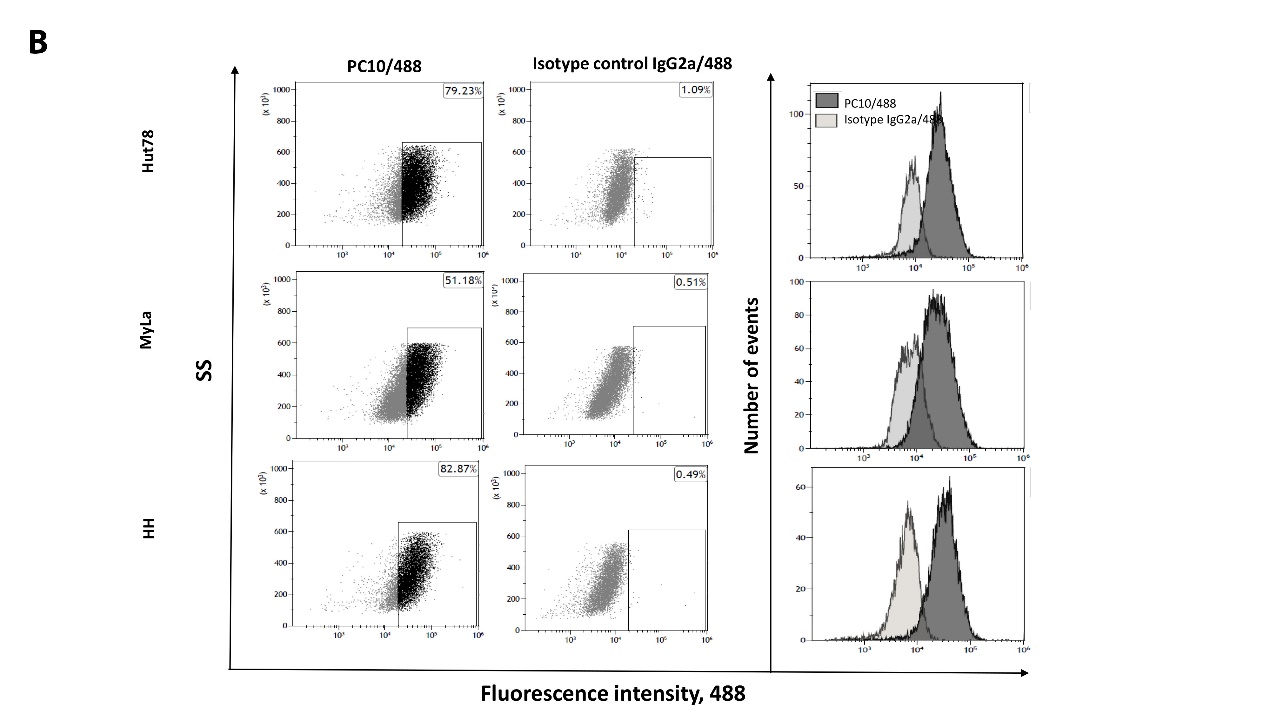


**Figure S1.** Staining with PC10 vs isotype IgG2a on un-fixed cells without permeabilization **(A)**, and on fixed and permeablized cells **(B)** n=3 experiments.

**
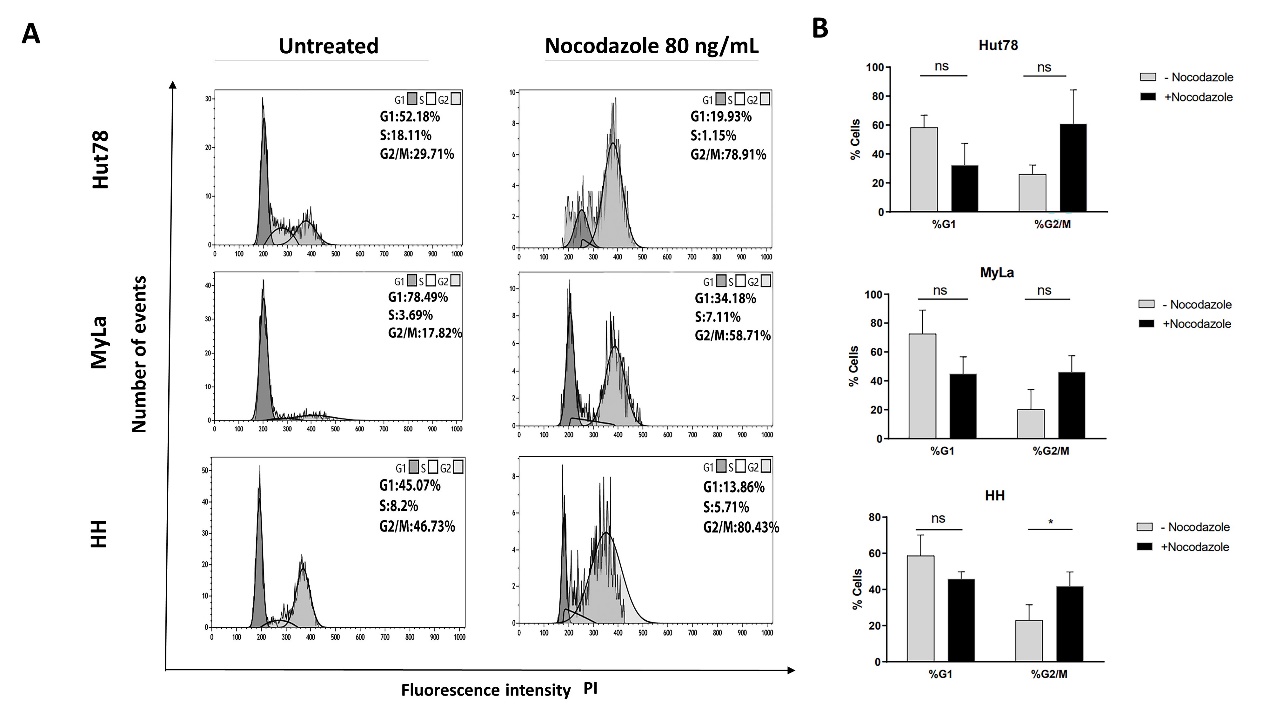
**

**Figure S2.** Cells were treated with Nocodazole (80 ɳg/mL 24 hr for Hut78, 100 ɳg/mL 48 hr for MyLa and 100 ɳg/mL 36 hr for HH) and their arrest in G2/M was confirmed by cell cycle distribution **(A),** the percent of G2/M arrest are shown in column curves, n=3 **(B).**
